# A method for studying pico to microsecond time-resolved core-level spectroscopy used to investigate electron dynamics in quantum dots

**DOI:** 10.1038/s41598-020-79792-z

**Published:** 2020-12-31

**Authors:** Tamara Sloboda, Sebastian Svanström, Fredrik O. L. Johansson, Aneta Andruszkiewicz, Xiaoliang Zhang, Erika Giangrisostomi, Ruslan Ovsyannikov, Alexander Föhlisch, Svante Svensson, Nils Mårtensson, Erik M. J. Johansson, Andreas Lindblad, Håkan Rensmo, Ute B. Cappel

**Affiliations:** 1 Division of Applied Physical Chemistry, Department of Chemistry, KTH Royal Institute of Technology, 100 44 Stockholm, Sweden; 2 Division of Molecular and Condensed Matter Physics, Department of Physics and Astronomy, Uppsala University, Box 516, 751 20 Uppsala, Sweden; 3 Department of Chemistry - Ångström Laboratory, Uppsala University, Box 523, 751 20 Uppsala, Sweden; 4 School of Materials Science and Engineering, Beihang University, Beijing, 100191 China; 5 Institute Methods and Instrumentation for Synchrotron Radiation Research, Helmholtz-Zentrum Berlin GmbH, Albert-Einstein-Straße 15, 12489 Berlin, Germany; 6 Institute of Physics and Astronomy, University of Potsdam, Karl-Liebknecht-Straße 24/25, 14476 Potsdam, Germany; 7 Uppsala-Berlin Joint Laboratory on Next Generation Photoelectron Spectroscopy, Albert-Einstein-Str. 15, 12489 Berlin, Germany

**Keywords:** Surfaces, interfaces and thin films, Quantum dots, Physical chemistry

## Abstract

Time-resolved photoelectron spectroscopy can give insights into carrier dynamics and offers the possibility of element and site-specific information through the measurements of core levels. In this paper, we demonstrate that this method can access electrons dynamics in PbS quantum dots over a wide time window spanning from pico- to microseconds in a single experiment carried out at the synchrotron facility BESSY II. The method is sensitive to small changes in core level positions. Fast measurements at low pump fluences are enabled by the use of a pump laser at a lower repetition frequency than the repetition frequency of the X-ray pulses used to probe the core level electrons: Through the use of a time-resolved spectrometer, time-dependent analysis of data from all synchrotron pulses is possible. Furthermore, by picosecond control of the pump laser arrival at the sample relative to the X-ray pulses, a time-resolution limited only by the length of the X-ray pulses is achieved. Using this method, we studied the charge dynamics in thin film samples of PbS quantum dots on n-type MgZnO substrates through time-resolved measurements of the Pb 5d core level. We found a time-resolved core level shift, which we could assign to electron injection and charge accumulation at the MgZnO/PbS quantum dots interface. This assignment was confirmed through the measurement of PbS films with different thicknesses. Our results therefore give insight into the magnitude of the photovoltage generated specifically at the MgZnO/PbS interface and into the timescale of charge transport and electron injection, as well as into the timescale of charge recombination at this interface. It is a unique feature of our method that the timescale of both these processes can be accessed in a single experiment and investigated for a specific interface.

## Introduction

Time-resolved spectroscopy techniques have a unique potential in providing insights in charge carrier dynamics at material interfaces. It is particularly interesting to implement such techniques for the study of any type of device that relies on fast charge transport and charge transfer across interfaces, such as, for example, solar cells, transistors and light emitting diodes^1–6^. Understanding the charge transfer and charge dynamics in such materials is of great fundamental interest as well as essential for further improvement in real device applications. There have been many reports of ultrafast spectroscopy trying to reveal the details of charge carrier generation dynamics, electron–hole separation etc. Most of these studies included infrared, visible and UV laser pulses for triggering and probing by measuring fluorescence or absorption^7–9^. However, these methods generally only probe the valence states of materials, which are very often distributed over several different atomic sites. With the use of X-rays as probe pulses, it is possible to access element-specific information. Time-resolved photoelectron spectroscopy (TRPES) using an ultraviolet, visible or near-infrared pump laser can be used to study the electron dynamics through a particular core level of the sample relating to a specific element and chemical state of the sample. By carrying out measurements at different time delays between the arrival of the laser (pump) and X-ray (probe) pulses on the sample, it becomes possible to monitor changes in core level peak positions and intensities over time and follow the kinetics of the processes causing these changes^6,10–15^.

Most frequently, this technique has been used so far to study photo-induced voltages between a sample surface and its bulk, i.e. to monitor the surface photovoltage (SPV) within the space charge region of semiconductor interfaces. The SPV effect arises, when the electrons and holes move away from/towards the surface (depending on the type of semiconductor) after excitation across the bandgap. This creates a small electric field which reduces the initial band bending and shifts the valence band maximum together with the core levels in the system^15^. Such shifts can be followed by photoelectron spectroscopy (PES) through the shifts of the core level binding energies (E_B_). However, a photo-induced voltage and related shift in the core level positions can also be caused by charge separation across an interface in a heterostructured sample. The timescales of such charge separation can be very different (longer) than the timescales in which the SPV effect is typically observed. Valuable information can therefore be gained in a measurement, where a wide time window is accessible. This can be achieved by carrying out time-resolved PES measurements at synchrotron radiation facilities, if an external pump laser is synchronized to the X-ray pulses generated by the synchrotron. The available time window is then limited by the pulse length of the X-rays and the accuracy of controlling the pump-probe delay for fast timescales and by the pump laser repetition rate for slow timescales. Furthermore, a time-resolved detection scheme is needed to assign electrons to specific X-ray pulses. Here, we present such TRPES measurements on lead sulphide quantum dot samples accessing a time window spanning six orders of magnitude, where we used the LowDosePES end-station at the synchrotron BESSY II. This was made possible through three factors: (1) The use of BESSY II in single bunch mode operation; (2) the use of an angle-resolved time-of-flight (ArTOF) analyzer for electron detection, which gave both time resolution and high transmission for electron detection and (3) a synchronized external pump laser operated at a much lower repetition frequency than the synchrotron.

The quantum dots (QDs) studied here are of interest among chemists and physicists, mainly because of the possibility of tuning the bandgap, but also for the high absorption coefficients, and the possibility of multiple-exciton generation in QD materials. Furthermore, QDs can be synthesized and processed from solution, enabling easy variations in the synthesis and therefore properties and making them economically accessible. In solar cells, QDs can be used in different architectures^16,17^. Our description here refers in particular to QD solar cells using a thin film of PbS quantum dots as the absorbing material. During past years, significant achievements were obtained regarding the stability and power conversion efficiency of solar cells based on this active material, due to immense research of their surface chemistry, charge dynamics, and energy band engineering. In particular, the solar cell performance has been improved through developments in surface passivation by different ligands^18–22^, cell architecture changes^23–26^, and modifications of the electron transporting layer^27–29^.

**Figure 1:**
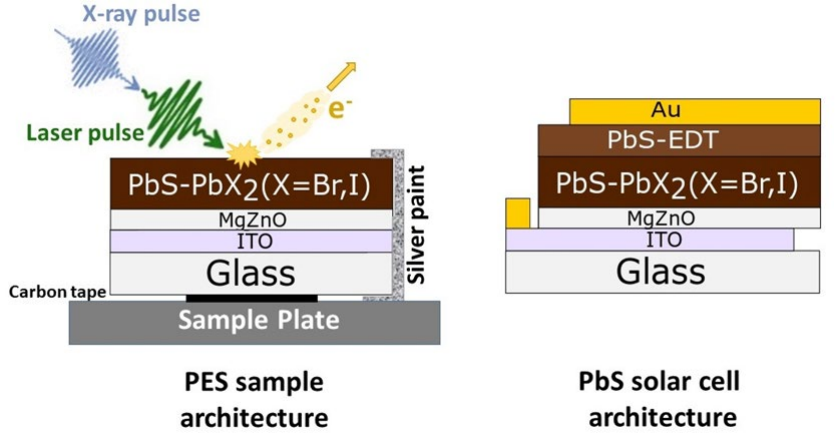
Architecture of QD samples used in photoelectron spectroscopy (left). The sample is connected to a metal sample plate through carbon tape and is grounded by applying silver paint. Architecture of a PbS-QD solar cell, which also includes a quantum dot layer with ethanedithiol ligand (EDT) and a gold contact (right).

In a typical solar cell architecture, an n-type metal oxide such as TiO_2_ or ZnO on a conductive glass substrate is used as the bottom layer for the solar cell structure (Fig. 1). This is followed by an n-type PbS quantum dots layer with halide ligands of a typical thickness of a few hundreds of nanometers followed by a thinner p-type PbS QD layer (typically with ethanedithiol (EDT) as the ligand) and a gold back contact. Further improvement to the cell performance is still achieved through an optimization of the ligand exchange for the n-type PbS QD layer. The highest reported efficiencies of PbS QD solar cells are now over 12%^22,26,30–32^, for quantum dots films prepared using the solution phase ligand exchange method^33^. In these solar cells, most light is absorbed in the thick n-type PbS QD layer and electrons have to be collected in the n-type metal oxide and holes in the p-type PbS QD layer. Charges can move from quantum dot to quantum dot through a hopping mechanism^34^. This charge transport has to be faster than the recombination of charges within the quantum dot layer and at contacts for efficient solar cells. Charge transport and charge recombination in complete solar cells are typically studied through charge mobility and photovoltage decay measurements^29,35,36^. In the measurements by TRPES presented here, we are able to study the charge transport and charge recombination between the metal oxide contact and the quantum dot absorber layer specifically within one measurement. This method is therefore complimentary to experiments carried out on complete devices. Using the quantum dot system as an example, we show how to carry out measurements and how to process the large amounts of data generated to obtain kinetic information at timescales covering six orders of magnitude. We demonstrate the advantages and flexibility of carrying out time-resolved measurements at a synchrotron: the high repetition frequency of the X-ray probe pulses reduces measurement times, while the variable repetition frequency of the laser pulses allows an optimized match to the time scales of the studied processes.

## Results and discussions

### Time-structure of experiments

In our time-resolved PES experiments, the X-rays generated by the synchrotron are used as the probe with a given repetition frequency, *f*_X_. For measurements carried out with the ArTOF spectrometer available at the LowDosePES end-station, *f*_X_ is the single bunch repetition frequency of Bessy II of 1.25 MHz. At this end-station, single bunch X-ray pulses can be obtained, not only when the measurements are carried out in single-bunch operation of the synchrotron, but also during the most common hybrid-bunch operation mode by selecting the camshaft bunch with the chopper available at the PM4 beamline^37,38^. Through other operational modes of the synchrotron, an even higher *f*_X_ could be used for measurements, for example during the so-called 4-bunch mode, where 4 equally spaced electron bunches are injected in the ring and *f*_X_ becomes 5 MHz.

**Figure 2:**
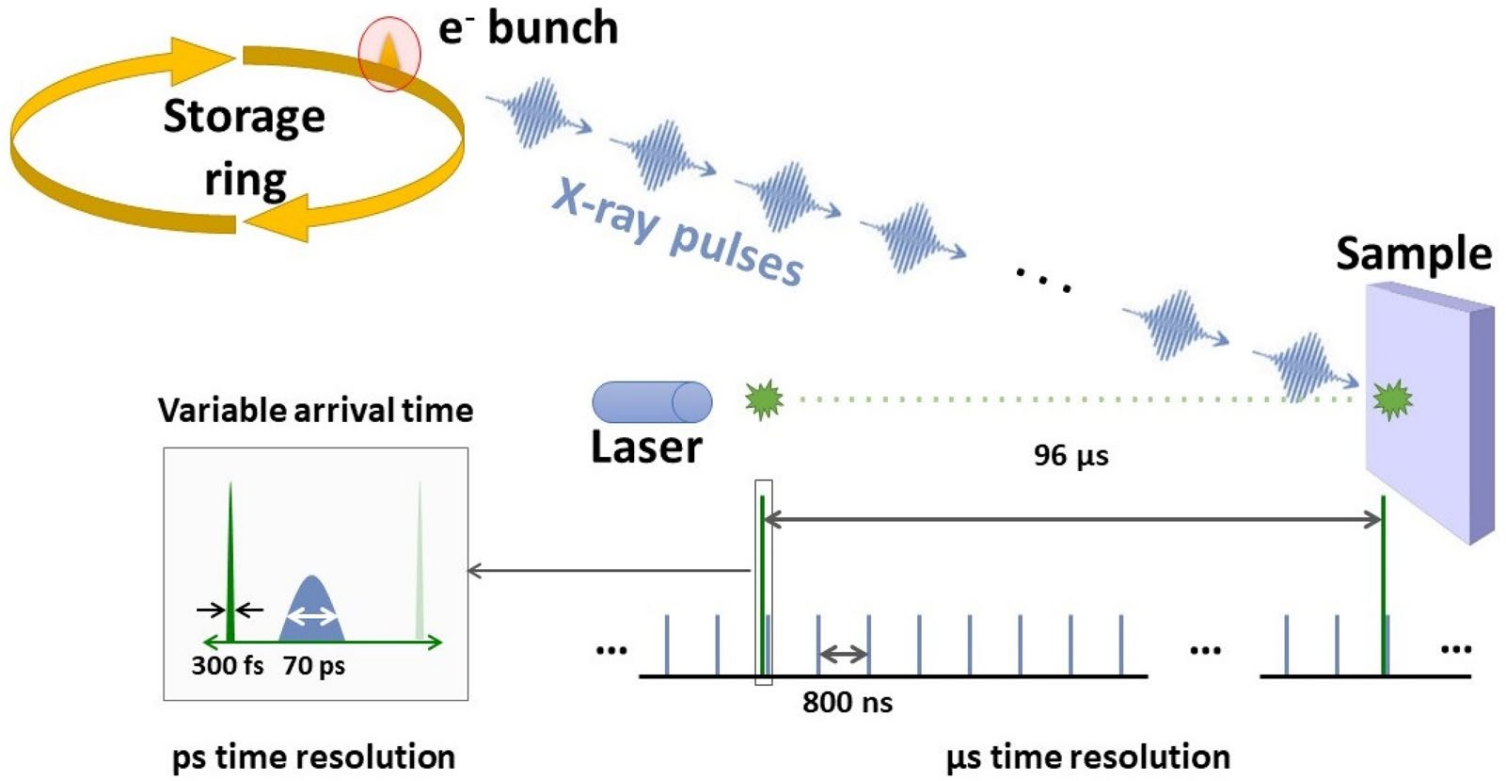
Time structure of pump-probe PES experiments at the LowDosePES end-station at BESSY II. Single bunch mode means that only one electron bunch is circulating in the storage ring, which passes the beamline dipole every 800 ns, generating X-ray photons that hit the sample. The pseudo single-bunch mode can be achieved during standard ring operation using the MHz chopper systemavailable at the end-station^37^. Meanwhile, the laser pulse arrives at the sample every 96 µs at the repetition rate chosen for this experiment. There are therefore 120 X-ray pulses between two laser pulses, giving the possibility of measuring 120 spectra at different pump-probe delay times for a fixed laser arrival time. Shorter pump-probe delay times can be measured by varying the arrival time of the pump laser in relation to the X-ray pulse train (bottom left inset).

The visible or NIR pump pulses are generated by a separate laser system with a variable repetition frequency *f*_L_. This repetition frequency should be an integer fraction of *f*_X_ such that: *f*_L_ = *f*_X_/*N*. In an experiment, we therefore have *N* X-ray pulses per laser pulse (Fig. 2). By analyzing the spectra generated by each of these X-ray pulses individually, a time-resolved measurement with a time range from 1/*f*_X_ to 1/*f*_L_ can be carried out. The spectral map determined from such an analysis will be referred to as “trigger time map” in this paper. In the time-resolved measurements presented below, *N* was 120 and therefore a time range from 800 ns to 96 μs, with a time step of 800 ns, was accessible for a measurement with a fixed laser arrival time.

A smaller time step can be achieved by varying the exact arrival time of the laser pulses in relation to the X-ray pulses with picosecond precision (Fig. 2). Such an experiment will be referred to as a “delay scan” in this paper. The time resolution of the experiment is then limited by the pulse lengths of pump and probe and by the precision in their relative arrival times. In the case of single-bunch mode at BESSY II, this is mostly dominated by the electron bunch length of approximately 70 ps (full width at half maximum), while the laser pulse length is sub-ps (≈ 350 fs for the results shown below)^37^. While it is possible to use a linear time step similar to the time resolution of the experiment for delay scans close to t_0_, we found such an approach unpractical (due to an excessive number of time points) to bridge the large time gap between two consecutive X-ray pulses (800 ns). Instead, we used a logarithmic stepping of the pump-probe delay times enabling us to effectively and continuously cover sub-ns to microsecond timescales in a single experiment. In the measurements presented here, we used 15 delay points between 100 ps and 400 ns with a logarithmic distribution on the time-axis. By combining delay scans with varying arrival times with the separate analysis of all X-ray pulses associated with one laser pulse, we were able to carry out experiments over a wide time window from 10^−1^ to 10^5^ ns, i.e. over 6 orders of magnitude.

### PbS quantum dots

In this study we investigated thin films of lead sulfide quantum dots with lead iodide/bromide ligands in a 5:1 molar ratio (referred to PbS-PbX_2_). The quantum dot film is deposited from a highly concentrated quantum dot ink already containing the ligand-exchanged quantum dots on indium-doped tin oxide substrates (ITO) coated with a thin layer of magnesium-doped zinc oxide (MgZnO), which serves as a transparent n-type selective contact for electron extraction in the solar cell (Fig. 1). In a complete solar cell, an additional thin layer of PbS QDs coated with 1,2-ethanedithiol (EDT) molecules as ligands is deposited as a p-type hole-selective layer and a thin gold layer as a contact (Fig. 1). As previously explained, such a device structure can give power conversion efficiencies exceeding 12%^30–32^.

UV–visible absorption spectroscopy of the PbS QD films with lead iodide/bromide ligands shows an exciton peak at about 938 nm, whereas the oleic acid-capped PbS quantum dot (PbS-OA) octane solution (the precursor solution) shows an exciton peak at 912 nm (supporting information, Figure S1). The bandgap of the quantum PbS QD is estimated to be 1.3 eV from the absorption maxima corresponding to a quantum dot size of approximately 3–4 nm^20,39^.

**Figure 3:**
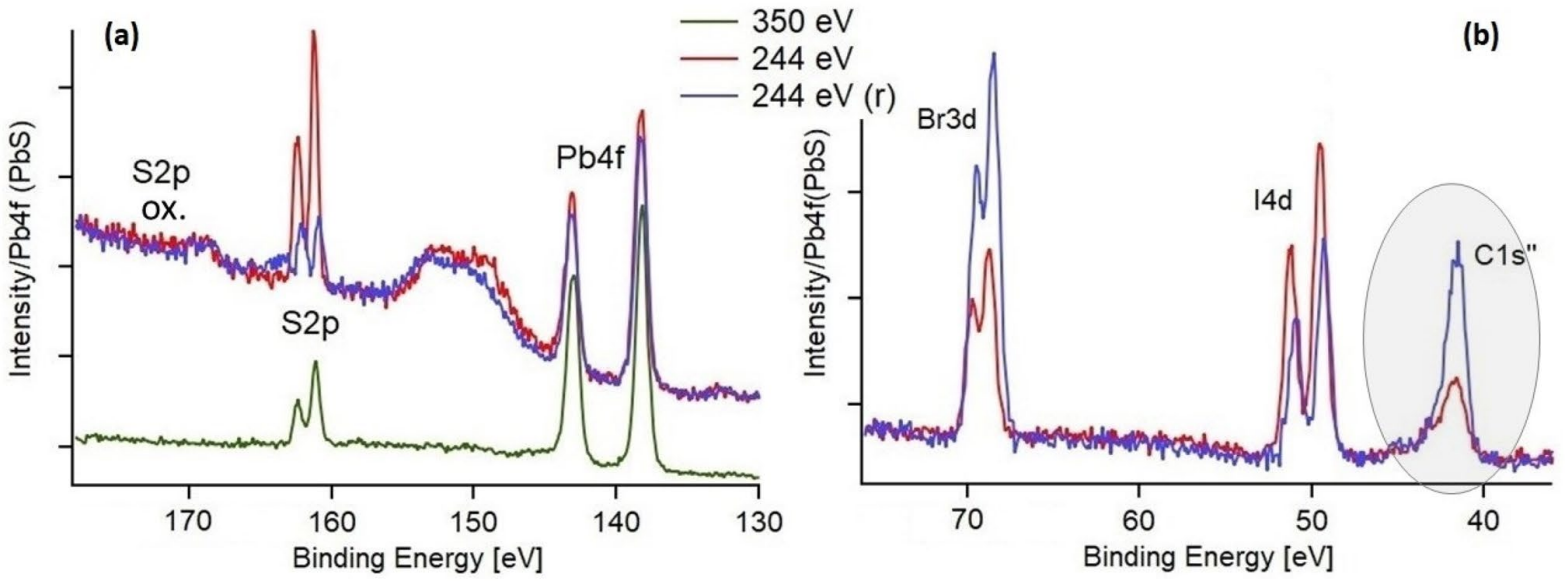
Steady state PES spectra of 250 nm thick PbS quantum dot sample at 244 and 350 eV photon energy. 244 eV (r) shows a measurement of a sample which had been exposed to ambient atmosphere for several days. (**a**) Pb 4f and S2p region. The wide peak at 150 eV is an Auger peak from Pb. (**b**) I 4d and Br 3d region. The C 1 s peak observed originates from the 2nd order of light refracted from the monochromator (488 eV).

Before pump-probe measurements the sample surfaces were characterized with steady-state PES using photon energies of 350, 244 and 90 eV at the LowDosePES station. Measurements with 350 eV show a single sulfur species, which has been previously assigned to sulfur in PbS^20^ (Fig. 3a), while the highly surface sensitive measurements with 244 eV show some additional sulfur species. The relative amount of the oxidized species varied between different parts of the sample and increased over time with storage in ambient atmosphere, whereas the main S 2p peaks decrease with the storage time (blue trace in Fig. 3a and curve fits of this data in supplementary information with Table S1 and Figure S2). The Pb 4f spectra could be fitted to two doublets of which the main one at lower binding energies is assigned to lead–sulfur and lead-iodide bonds (Figure S3). We also observed some variation in the amount of bromide, iodide and carbon present at the sample surface (Br 3d, I 4d and 2nd order C 1 s peak in Fig. 3b). The Br and C quantity increased in the sample measured after being exposed to ambient atmosphere for a few days, whereas the I quantity decreased, which is in agreement with our previous measurements^40^.

We also investigated the bulk quality of duplicate samples at the GALAXIES beamline at SOLEIL synchrotron with a photon energy of 3000 eV. Due to the higher photon energy, we were able to probe more towards the bulk of the samples, where the results were less influenced by surface contamination. The measurements showed the single sulfur species assigned to PbS QD and quantification revealed a S/Pb ratio of approximately 1:2 in the samples (Figure S4 and Table S2 in supporting information). These results suggest that, while some chemical differences can be observed for sulfur species at the sample surface, the samples typically have a high bulk quality. Furthermore, at the LowDosePES end-station, we had high count rates in measurements of Pb 5d with a photon energy of 90 eV suggesting that surface contamination is not severe enough to compromise signal intensities. No changes in the surface chemistry were observed through the exposure of the samples to the pump laser.

### Pump-probe measurements

**Figure 4:**
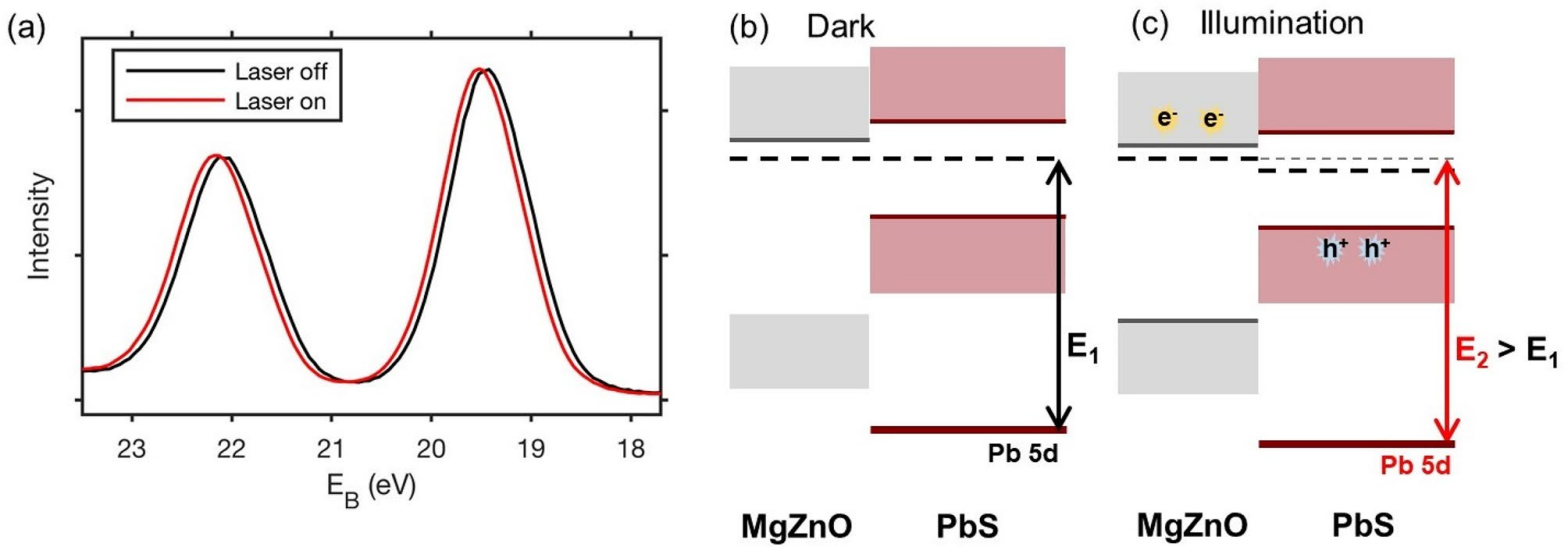
(**a**) Pb 5d spectra of a 250 nm thick PbS quantum dot sample measured with a photon energy of 90 eV for 180 s without and with laser illumination with an average power of 0.16 mW. (**b**) Schematic energy diagram of the sample in the dark: The Fermi levels of the MgZnO substrate and of the PbS quantum dots are aligned. The binding energy of the Pb 5d core level is defined as E_1_. (**c**) Schematic energy diagram of the sample during illumination: electron injection into the MgZnO substrate leads to quasi-Fermi level splitting. The PbS QD layer becomes positively charged and the Pb 5d core level is observed at a higher binding energy (E_2_, defined as the energy difference between the Pb 5d level at the surface and the Fermi level of the substrate). This simple model does not show possible band bending at the interface, as this cannot be directly observed in the measurements.

For measurements with the pump laser, we measured the Pb 5d core level with a photon energy of 90 eV due to the high signal intensity and energy resolution with the used beamline and spectrometer settings. Figure 4a shows a spectrum measured during laser illumination with a laser power of 0.16 mW and *f*_L_ = 10.4 kHz compared to a spectrum measured without laser illumination on the same sample spot. In the case of laser illumination, the delay time between the laser pulse and the first X-ray was set to 100 ps, but the spectrum was obtained from all 120 X-ray pulses, which hit the sample (in between two laser pulses), and therefore gives an average spectrum during laser illumination. For both spectra, the ArTOF spectrometer was used in fixed mode with a center kinetic energy of 65 eV and the total measurement time was 180 s. A shift to higher binding energies is observed upon laser illumination. This shift can be explained through charge separation between the PbS quantum dots and the MgZnO substrate. In the dark, the Fermi level of the quantum dots and that of the substrate align (Fig. 4b). Upon illumination with the 515 nm laser, electron–hole pairs are generated in the quantum dot film. Some of the excited electrons will be injected into the n-type MgZnO substrate leading to a splitting of the quasi-Fermi levels between the MgZnO and the PbS QD layer (Fig. 4c). For any injected electron, a hole remains in the PbS QD layer leading to a positive charging of the quantum dot layer. At steady state this leads to shift of all core levels of the PbS QD layer to higher binding energies, shown here for the Pb 5d level, i.e. to the generation of a photovoltage. In this model, the magnitude of the shift therefore depends on the number of injected electrons as well as on potential changes in band alignment at the interface, which accompany electron injection. Previously, a change in the band bending at the interface has been used to explain a time-resolved voltage change in the interface of a mono-layer of quantum dots with a ZnO single crystal^11^. In our measurements, we cannot directly observe the interface between the MgZnO and the quantum dots and can therefore not directly observe changes in band bending either. The schematic diagrams in Fig. 4b,c are therefore drawn without accounting for band bending. By using time-resolved PES measurements, we are able to follow the kinetics of the generation and the decay of the binding energy shift (see below).

### Analysis of trigger time maps and delay scans

**Figure 5:**
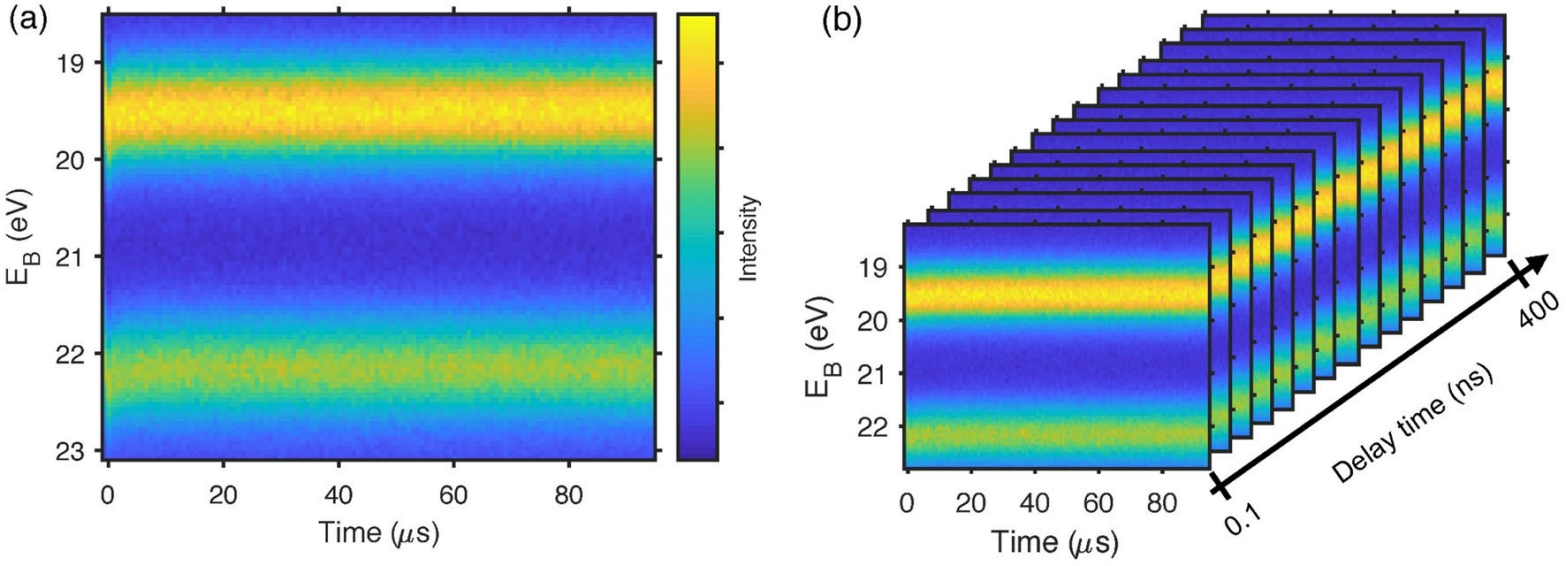
Visualisation of the data obtained in TRPES measurements: (**a**) Trigger time map of the Pb 5d core level measured at a delay time of 122 ns for a 250 nm thick PbS quantum dot sample with a laser pulse energy of 15.4 nJ and a probe energy of 90 eV. (**b**) Visualization of a delay scan on the same sample. Trigger time maps are obtained at 15 different pump-probe delay times.

Figure 5a shows an example of a trigger time map of the Pb 5d core level of a PbS quantum dot sample measured with a laser power of 0.16 mW (15.4 nJ per pulse and 4.0 · 10^10^ photons per pulse) and *f*_L_ = 10.4 kHz. Through a measurement of the spot size, this was estimated to correspond to a laser fluence of 9.5 μJ cm^−2^ per pulse (see experimental details). As described above, such a map spans a trigger time region of 1/*f*_L_ (= 96 μs in this case). A shift in the core level position can be observed at times below 20 μs. Figure 5b shows the complete spectral data set for a delay scan of the same sample: A trigger time map is obtained at each laser to X-ray delay time ranging from 0.1 to 400 ns with a logarithmic step between the delay points.

It is clear that delay scans carried out in this way generate a large number of spectra, e.g. 120 spectra per delay time × 15 delay times = 1800 spectra in a logarithmic delay scan. We therefore employed a simplified curve fitting routine to extract parameters by fitting the Pb 5d spectra with two Gaussian peaks and a polynomial background. The polynomial background was used as it was found to best model the detector background obtained in a fixed mode energy measurement with the ArTOF spectrometer.

**Figure 6:**
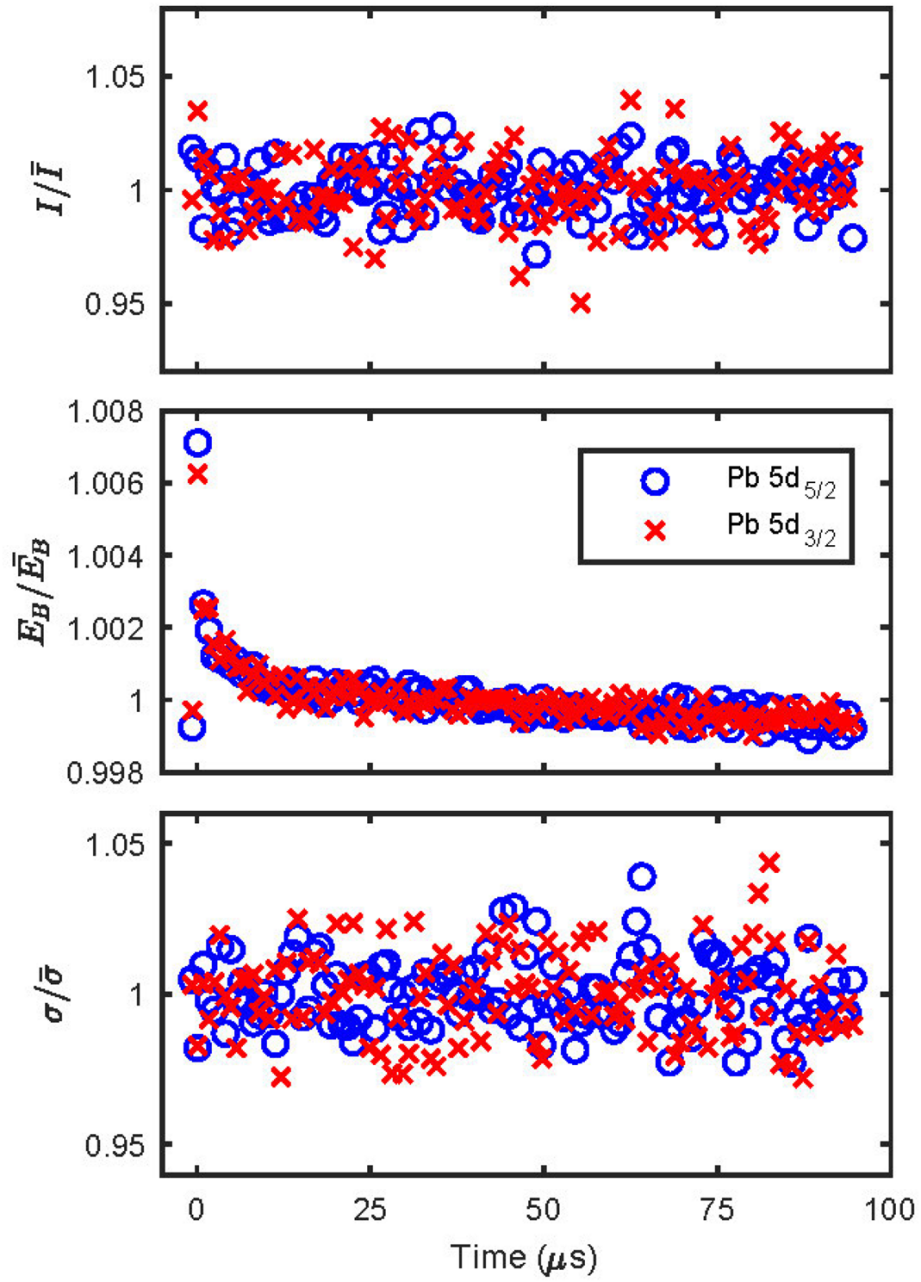
Fit parameters of the trigger time map shown in Fig. 5: top—intensity (I), middle—position (E_B_), bottom—width (*σ*). The parameters for the Pb 5d_5/2_ and Pb 5d_3/2_ are normalized to their arithmetic mean values over the whole trigger time map.

Figure 6 shows the peak position (E_B_), intensity (I) and width (*σ*) results of such a fit for the trigger time map shown in Fig. 5 normalized to the arithmetic mean values of each parameter for the whole trigger time map. No obvious time-resolved behavior is observed for the intensity (I) and the width (*σ*), while a clear time-resolved variation is observed in the peak position (E_B_), which follows the same trend for both Pb 5d peaks. Further analysis will therefore focus on the peak position.

**Figure 7:**
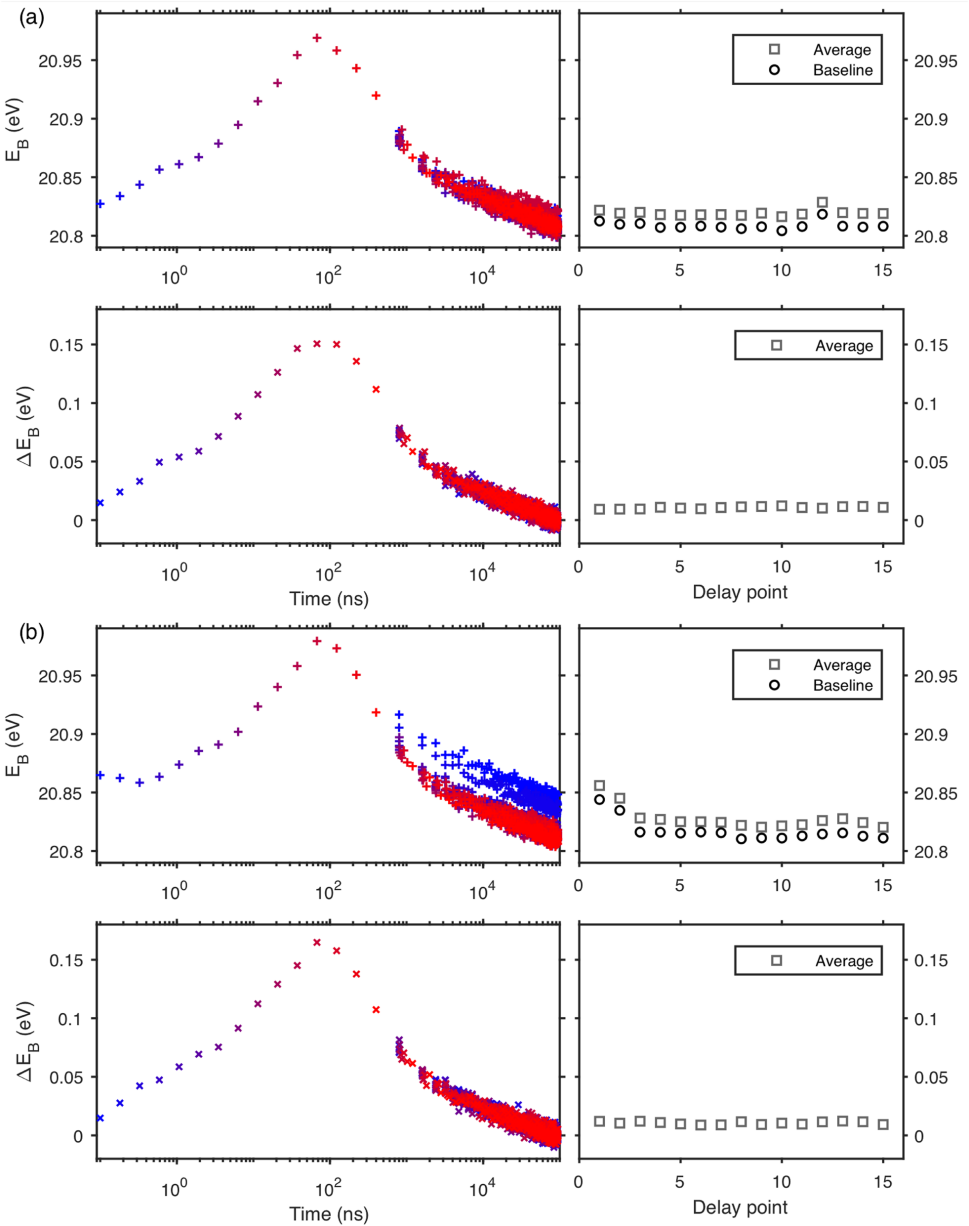
Examples of two delay scans in two different sample spots with a laser pulse energy of 15.4 nJ on a 250 nm thick PbS quantum dot sample presented in panels (**a**) and (**b**). The top left panel of each sub-figure shows the absolute peak positions (average of Pb 5d_3/2_ and Pb 5d_5/2_) obtained from fits with different colours representing results from trigger time map measured with different laser-X-ray delay times. The top right panel shows the average and baseline binding energy values calculated for the different trigger time maps, see text. The bottom left panel shows the peak positions with the baseline subtracted for each trigger time map. The bottom right panel shows the average relative binding energy shift.

**Figure 8:**
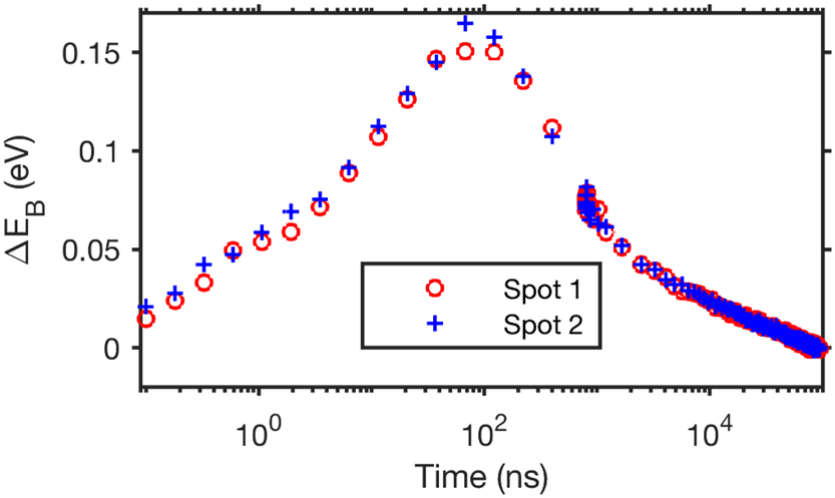
Comparison of averaged delay scans from Fig. 7 measured in two different sample spots with a laser pulse energy of 15.4 nJ on a 250 nm thick PbS quantum dot sample. The fit parameters from the different trigger time maps were averaged from the 3rd X-ray pulse after the laser pulse onwards (approximtely from 1600 ns).

Analysis of all X-ray pulses associated with one laser pulse leads to a large redundancy of data when recording delay scans with varying pump-probe arrival times. This data can on one hand be used to improve the signal-to-noise of delay scans in the microsecond time regime but on the other hand also to check sample stability during delay scans. Figure 7 shows two examples of this. Panels (a) and (b) show the fit position averaged over the two spin–orbit-split peaks from two delay scans carried out on two different spots of the same sample with the same laser pulse energy. For each delay scan, the peak positions obtained directly from the fit (average of Pb 5d_5/2_ and Pb 5d_3/2_ position) are plotted as a function of time on a logarithmic scale in the top left figure. Each color in the graph (in total 15 colours going from blue to red) represents the results of the analysis of a separate trigger time map at a different laser delay (i.e. one panel in Fig. 5). Due to the logarithmic time-axis, the data points from different delay times are seen as clearly separated for the first X-ray pulse. However, for the later X-ray pulses, the data points collapse on top of each other, as the difference in delay time becomes small compared to the trigger time difference between the laser pulse and the X-ray pulses. We also calculated an average binding energy position (Ē_B_) for each trigger time map by evaluating the average of all E_B_ positions for the trigger time map. The Ē_B_ can give a good indication of overall variations between consecutive measurements, which extend beyond variations due to a change in delay time. The Ē_B_ parameters for each trigger time map, as well as a baseline value calculated from the last 15% of the data points in a map (a time region, where there is only insignificant time-resolved change in the binding energy positions) are displayed on the right in Fig. 7. In Fig. 7a, a small variation in these parameters is observed (the standard deviation of the average is 2.9 meV), while in Fig. 7b there is a clear difference in the absolute spectral positions of the first two measurements. The standard deviation of the average is 9.8 meV in this case. In the bottom left panels of Fig. 7a,b, E_B_ values are shown, where the baseline values of the corresponding trigger map have been subtracted. The resulting relative binding energy shifts at long time scales collapse on top of each other for both data sets and the standard deviation of the average values becomes 1.4 and 1.5 meV for the data in Fig. 7a and b respectively. This indicates that the variations observed in the data presented in Fig. 7b are related to the absolute peak positions but do not influence the kinetic traces as much. In this case the fit parameters obtained for later X-ray pulses can be averaged to improve the overall signal-to-noise ratio of the data. Figure 8 shows the averages obtained for the data in Fig. 7, where the fit parameters were averaged for the different delay points from the 3rd X-ray pulse onwards (approximtely from 1600 ns onwards). The standard deviation of the fit values from different delay point measurements is approximately 4 meV. A good match of the kinetic traces obtained from the two different sample spots is observed. Furthermore, a very clear time-resolved signal is observed, despite the use of a relatively low pump fluence for a time-resolved experiment (15.4 nJ per pulse is estimated to be about 9.5 μJ cm^−2^ per pulse) and a relatively short measurement time (the total measurement time for one complete delay scan was roughly 45 min, meaning that about 90 min of measuring was needed for the two delay scans shown in Fig. 8). Obtaining such signals was enabled through the measurement of core levels at a synchrotron light source in the current study and would not be possible with either a low frequency X-ray or a UV source. While it is possible to achieve similar photon energies to the one used here in lab-based sources, this still comes at a sacrifice of repetition frequency^41^. In the future, it may become possible to use a similar methodology at free electron lasers^42,43^ or lab-based, time-resolved X-ray sources^41^ and the development of TRPES will benefit from the development of sources.

### Impact of laser power and quantum dot film thickness on kinetics

**Figure 9:**
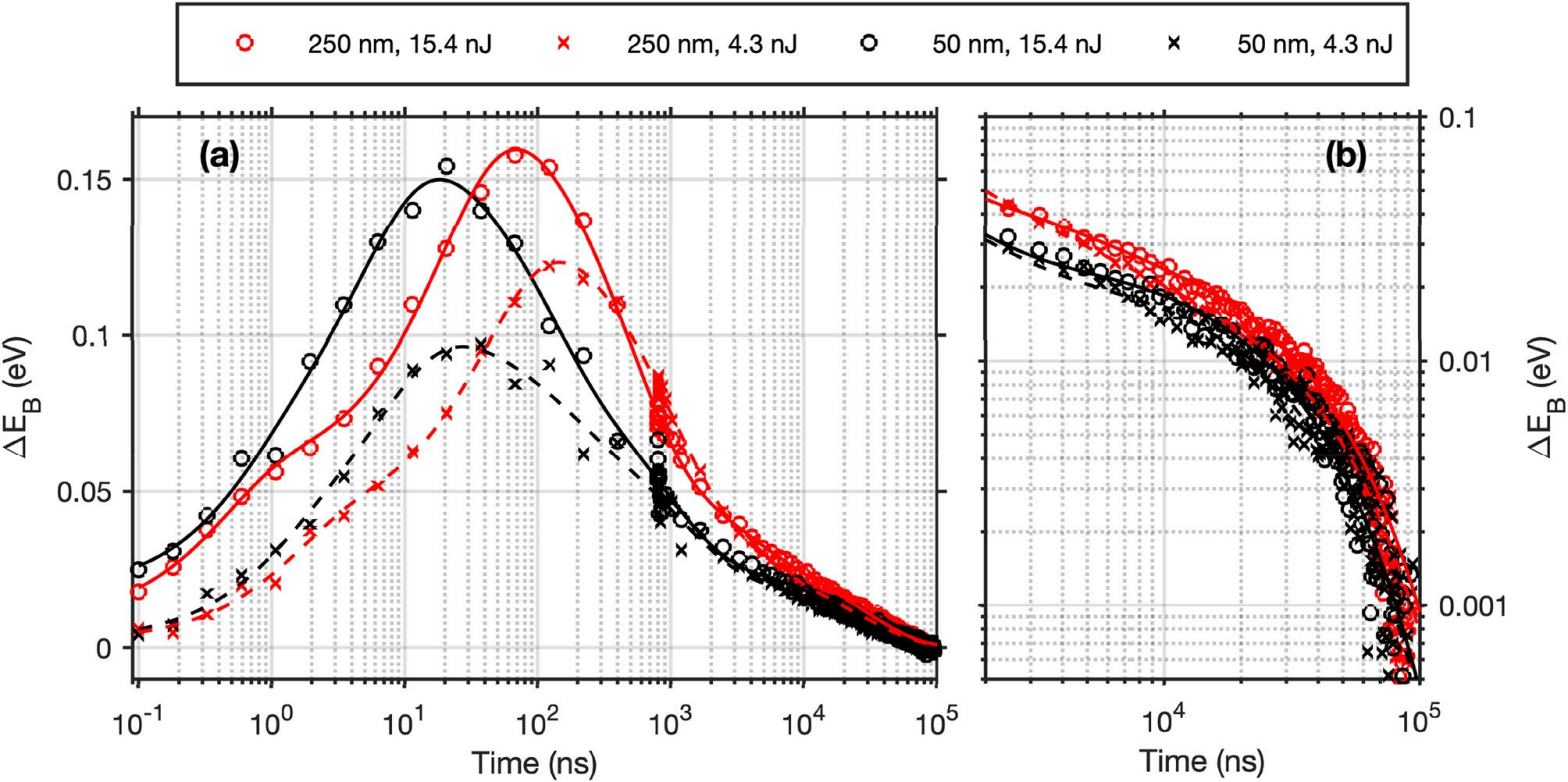
Kinetic traces obtained from delay scans on two PbS quantum dot samples with different thicknesses (250 nm, red and 50 nm, black) at two different laser pulse energies (15.4 nJ , circles and 4.3 nJ , crosses). Multi-exponential fits to the data are included as a guide to the eye. (**a**) Complete time range, (**b**) focus on longer times (> 2 µs) of the same delay scans with both axes on a logarithmic scale.

We now turn to the impact of laser power and the quantum dot film thickness on the time-resolved binding energy changes. Applying the same analysis as described above to delay scans measured on two PbS QD samples with different thicknesses with laser pulse energies of 15.4 nJ and 4.3 nJ per pulse resulted in the kinetic traces presented in Fig. 9. All traces first show a shift of the Pb 5d core level to higher binding energies and then back to the original binding energy over the time scale of the measurement, i.e. the generation and decay of a photo-induced voltage across the sample. Multi-exponential fits are included in Fig. 9 as a guide to the eye and to give an estimation for the timescales of the photovoltage generation and decay. Five exponential time constants were required—two for the rise and three for the decay. Details of the equation and fitted parameters are presented in the supplementary information (Table S3). Multiple exponential decays are often used to model the results in time-resolved voltage measurements in these type of systems^44,45^. However, we would like to highlight that at present, there is no scientific model underlying our exact fitting procedure and instead it was used to extract average time constants used for comparison. Additionally, the maximum photovoltages and the times, at which they occur, were extracted and are presented in Table 1.

**Table 1:** Maximum photovoltage (*V*_max_) and delay time at which the maximum photovoltage was observed (*t*_max_) of kinetic traces obtained for the 250 nm and the 50 nm thick PbS samples at laser pulse energies of 15.4 and 4.3 nJ.

PbS thickness/nm	250	250	50	50
Laser pulse energy/nJ	15.4	4.3	15.4	4.3
*V*_max_/eV	0.160	0.123	0.150	0.096
*t*_max_/ns	69	145	18	27

**Figure 10:**
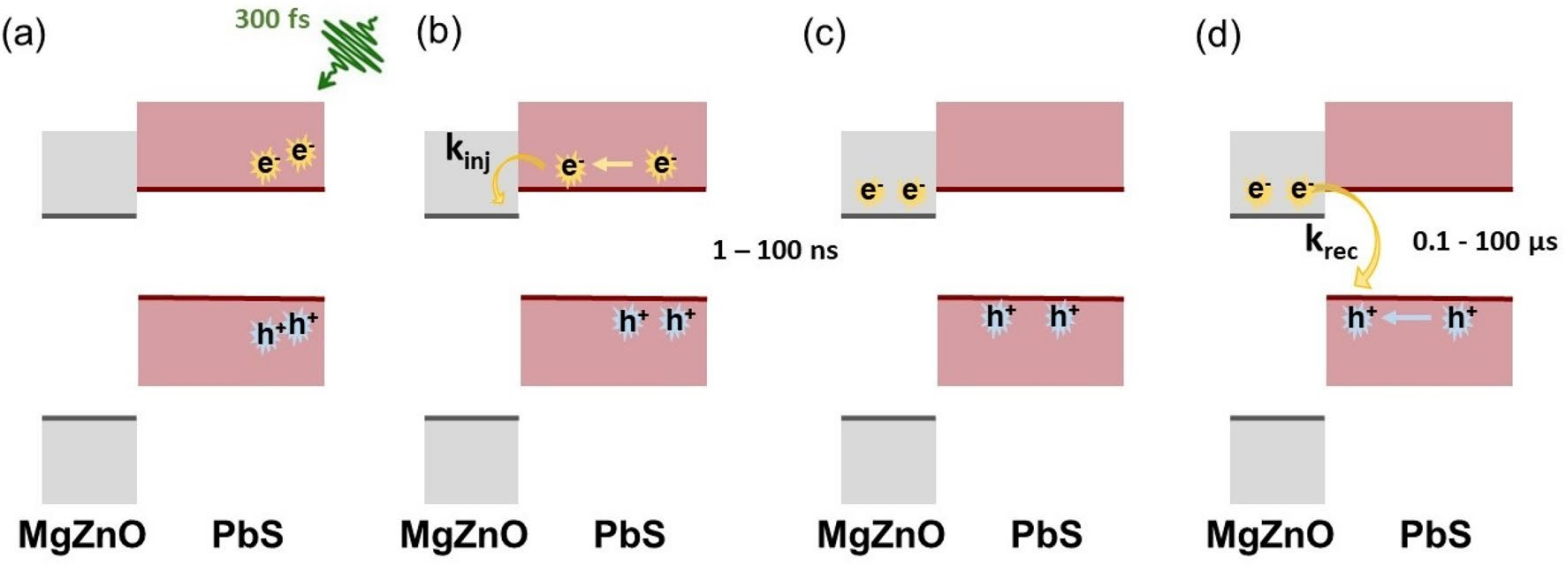
Scheme of electron dynamics occurring between two laser pulses at the MgZnO/PbS QD interface. (**a**) Interaction of the laser pulse and PbS QD layer leading to the creation of electron–hole pairs. (**b**) Diffusion of electrons and holes followed by electron transfer to the MgZnO. (**c**) Accumulation of electrons in the MgZnO layer. This is observed by the increase in ΔE_B_. (**d**) The system goes back to the equilibrium state, when electrons and holes move to the boundary, where they recombine with a rate constant of *k*_rec_. This process is observed by a decrease in ΔE_B_.

Reducing the laser pulse energy leads to a smaller absolute magnitude of the E_B_ shift, and the highest photovoltage is also being reached at a slightly longer delay time (Fig. 9 and Table 1). The decay of the photovoltage however happens with a similar rate for both powers investigated. In contrast to this, decreasing the film thickness leads to both a faster rise and a faster initial decay of the photo-induced binding energy shift, but not to a significant decrease in magnitude. The average fitted rise time is 4.0 ns for the 50 nm thick PbS QD film, while it is 16 ns for the 250 nm thick film at 15.4 nJ per pulse. The average decay time increases from 4400 ns for the 50 nm film to 5800 ns for the 250 nm film at the same laser pulse energy. These observations agree well with a model where the photo-induced shift is explained by charge separation from the quantum dots to the substrate. The charge injection from PbS quantum dots into ZnO and MgZnO is well documented in various studies^11,44–46^. Figure 10 shows a scheme of a model of the processes proposed to occur for our PbS QD/MgZnO interface between two laser pulses (roughly 96 µs, see Fig. 2). The processes are initiated by light absorption and the generation of electron hole pairs in the quantum dot film. The electrons in the QD film will then have to travel to the interface, where they can be injected into the MgZnO (Fig. 10b). The rate of generation of electrons into MgZnO substrate (*G*) can then be described as:

1 $$G={k}_{\mathrm{inj}} {n}_{\mathrm{I}}$$

where *k*_inj_ is the rate constant for injection and *n*_I_ is the number of electrons in the quantum dot layer at the interface with MgZnO. n_I_ will be time-dependent and depend on the number of absorbed photons, on the charge transport to the interface and the recombination within the quantum dot film. Recombination of electrons in the MgZnO is likely a 2nd order process (Fig. 10d) and its rate (*R*) is given by:

2 $$R={k}_{\mathrm{rec}} {p}_{\mathrm{I}}{n}_{\mathrm{S}}$$

where *k*_rec_ is the rate constant for recombination, *p*_I_ is the number of holes in the quantum dot layer at the interface with MgZnO available for recombination and *n*_S_ is the number of electrons within MgZnO substrate available for recombination. Overall, the rate of change in *n*_s_ is then given by

3 $$\frac{\mathrm{d}{n}_{\mathrm{S}}}{\mathrm{d}t}=G-R$$

Initially (at short delay times after the laser pulse) the rate of generation will be higher than the rate of recombination leading to an increase in the number of injected electrons and therefore also in the photovoltage. As the number of injected electrons increases, the rate of recombination will increase and the maximum number of charges and highest photovoltage will be observed at the delay time, where G = R (see Fig. 9 and Table 1). Following this maximum, the number of charges will decrease due to recombination and the kinetic traces will begin to mostly reflect the rate of recombination (Fig. 9b).

A higher laser pulse energy will increase the number of photons absorbed by the quantum dot film and therefore a higher number of charges are generated within the quantum dot film leading to more charge separation to the substrate and therefore to a higher photovoltage (Table 1). This is in agreement with our observation of complete solar cells based on the same substrate and quantum dot type^20^. If the recombination rate depends primarily on the charge concentration (Eq. 2), then the rate of recombination increases with the laser pulse energy, leading to this rate surpassing the generation rate at earlier delay times (Table 1). However, when the amount of charges (and therefore the photovoltage) has decreased to the same amount, the rate of recombination will be the same independent of the initial laser pulse energy. This is observed from delay times of approximately 1 μs onwards here (Fig. 9b). When discussing the different film thicknesses, it becomes important to consider the sample geometry: Samples are illuminated from the quantum dot side of the samples (Fig. 1) with a 45° angle between the laser and the sample surface. Light is therefore absorbed more strongly close to the top surface of the 250 nm quantum dot film (the penetration depth is estimated from the absorption spectrum to be about 76 nm for the 250 nm thick film) and charges are therefore mostly generated away from the MgZnO substrate. An increase in the film thickness therefore leads to a direct increase in how far charges have to travel before reaching the substrate and therefore to a change in *n*_I_ and *G* as a function of time (Eq. 1). A slower rise was observed for the thicker film compared to the thinner one showing that *G* depends on the film thickness and therefore on the charge transport time to the substrate. On the other hand, the decay in the photovoltage depends on the rate of recombination between electrons in the MgZnO and holes in the quantum dots. This process can depend on several factors including charge trapping in the MgZnO and the rate of hole diffusion to the MgZnO interface and can therefore show complex kinetics. By increasing the thickness of the PbS QD film, the volume over which the holes can spread increases, which could lead to a reduction in the recombination rate through a decrease of *p*_I_ according to Eq. (2).

When comparing our results to previous studies, one notes that there are few studies on PbS quantum dots, which investigate such a wide time range in a single measurement. A previous TRPES study focused on a PbS monolayer on ZnO with a time resolution of 150 ns, but used a continuous wave laser for excitation instead of a pulsed laser^11^. Both the difference in experimental set-up and in the sample lay-out means that the kinetics observed in their study cannot be directly compared to those observed in ours. A continuous wave laser means that the rise time of the photovoltage will correspond to the time it takes to reach an equilibrium between charge separation and recombination and not directly to the charge separation time. In their system charge transport between quantum dots is not expected to play a significant role, while in our case the rise time of the photovoltage clearly depends on the quantum dot film thickness. We note however that the photo-induced binding energy shift observed here is significantly larger (> 100 meV) than that observed in this previous study (< 30 meV).

Recombination dynamics of thin film quantum dot samples are often investigated via photovoltage decay measurements on complete solar cells and focus on the microsecond to millisecond timescales^29,44,45^. Typical voltage decay times for systems similar to ours (a thick PbS quantum dot layer with a MgZnO electron extraction layer, see Fig. 1) show recombination times ranging from tens of microseconds to milliseconds^20,44^, which is a significant difference to the recombination times ranging from hundreds of nanosecond to tens of microseconds observed here. This difference can have two main reasons: In the complete solar cell, further interfaces at which charge separation can occur are introduced – namely to a p-type quantum dot layer and from that to a metal contact. This can change the overall recombination time, and photovoltage decay measurements are not specific to particular interfaces in the solar cell. In contrast, our TRPES measurements focus on the interface between MgZnO and the PbS quantum dot layer. Furthermore, the difference observed in the binding energy of a sample fully in the dark (Fig. 4a) and at long delay times suggests that not all charges recombine fully within the timescale of our measurement. In contrast, in time-resolved photovoltage measurements fast components of the voltage decay cannot always be resolved within the time resolution of the experiments. Our measurements therefore focus on the faster component of the voltage decay occurring at the MgZnO/PbS interface in absence of a hole selective interface.

Laser-based time-resolved studies have often focused on ultrafast processes in PbS quantum dot systems either through investigating the dynamics of transient absorption or time-resolved photoluminescence^36,47^. In this way, the charge injection from PbS quantum dots to ZnO or MgZnO substrates has been studied and was found to occur on ultrafast timescales including sub-picosecond^44,46^. However, it should be noted that such studies have often been carried out on systems, where a mono-layer of the quantum dots is adsorbed to the metal oxide and charge transport through a quantum layer is not necessary^46^. This suggests that the photovoltage rise times observed here are mostly limited by the charge transport through the quantum dot layer and not by the injection into the substrate itself.

## Conclusions

In summary, we have shown that the set-up at the LowDosePES station at Bessy II can be used for time-resolved core level photoelectron spectroscopy with time scales spanning six orders of magnitude. The charge dynamics in PbS quantum dot films of relevance for solar cells could be investigated following the binding energy shifts in the Pb 5d core level induced by the visible pump laser. In particular, this type of measurement bridges the timescales investigated in ultrafast laser measurements and those in transient photovoltage measurements on complete solar cell devices and should in the future allow for a simultaneous investigation of charge separation and charge recombination processes. Such measurements were made possible through the combination of a highly efficient spectrometer, an X-ray photon energy optimized for high photoemission cross section and a pump laser with a variable frequency. By reducing the pump laser frequency, we were able to measure the dynamics of relevance to our system by analyzing all single-bunch X-ray pulses incident on the sample in between two successive laser pulses. The extra information available through the large number of X-ray pulses incident on the sample after one laser pulse also allows to assess sample stability during a delay scan measurement, where the arrival times of the laser at the sample is varied with picosecond precision in relation to the X-ray arrival time. The method presented is highly sensitive to even small core level shifts and good data quality was achieved with relatively short measurement times. Only moderate pump fluences were needed to be able to detect a time-resolved signal, which means that measurements with relevance to real systems can be carried out and damage of samples by the pump laser can be avoided.

The laser-induced binding energy shifts measure the build-up of a photovoltage in the sample, which we assign to charge separation with electron transfer to the substrate, accumulation and a subsequent recombination at the interface. The process is relatively slow compared to the build-up of a surface photovoltage, which is most commonly studied by time-resolved core level spectroscopy. Our interpretation is confirmed by the laser power and film thickness dependences of the observed kinetics. The measured photovoltage is specific to the interface studied (PbS/MgZnO in this case) and, in the future, the other interfaces in the solar cell such as the junction between n-type quantum dots with halide ligands and p-type quantum dots with ethanedithiol ligands could be investigated through targeted sample design.

By choosing the Pb 5d core level for analysis, we specifically investigated the energetics of the PbS quantum dots in this case. With the range of photon energies available at a synchrotron, core levels of other materials can also be studied, and this selectivity could be used in combination with sample design to investigate charge transfer between different materials.

## Experimental methods

### Materials

All reagents and solvents for the synthesis of the materials were purchased from Sigma-Aldrich. The MgZnO and the PbS QD particles were synthesized according to previously reported methods with small modifications^45,48^.

### Synthesis

The PbS QD synthesis begins with adding lead oxide (PbO, 99.99%, 0.933 g), oleic acid (OA, tech. grade 90%, 4.053 g), and 1-octadecene (ODE, tech. grade 90%, 25 ml) to a three-neck round bottom flask. The mixture is first degassed with nitrogen flow for 1 h and later heated to 100 °C and kept under nitrogen atmosphere for 2 h. Simultaneously, the hexamethyldisilathiane ((TMS)_2_S, synthetic grade, 0.356 g) solution in ODE (10 ml) was prepared in a glovebox. When taken out of the glovebox, the argon above the solution in the vial was carefully removed under fume hood with a syringe, leaving a mild vacuum condition inside the vial, which could then be moved into the oven and kept at 80 °C for another 2 h. After 2 h of stirring at 100 °C, the temperature of the reaction mixture was lowered to 90 °C and the (TMS)_2_S was quickly injected, giving rise to an instantaneous reaction. The heating source is removed 2–3 min after the injection, letting the PbS-OA particles cool down to room temperature. The newly formed PbS-OA were then cleaned in 2 washing steps with acetone and toluene, then dried under vacuum and finally dispersed in octane so that the concentration was between 80 and 40 mg/ml. The particles synthesized in this way have a diameter of about 3 nm with an optical energy gap of 1.3 eV.

### PES sample fabrication

The substrate used for sample fabrication was ITO-coated glass (1.1 mm ITO, ~ 12 Ω/sq), sequentially cleaned with diluted RBS-25 concentrate, acetone, and ethanol respectively in the ultrasonic bath and dried with air flow. Additionally, the clean ITO was treated with UV-ozone for 20 min directly before deposition of MgZnO nanoparticles. The MgZnO nanoparticles were filtered through a 0.2 µm filter and spin-coated on ITO-substrates at 3000 rpm for 30 s, followed by annealing at 200 °C for 30 min and 300 °C for another 30 min under fume hood. The PbS QD films were prepared according to a previously reported method with small modifications^49^. The starting concentrated PbS QD solution in octane was diluted to 10 mg/ml before being mixed with previously prepared ligand solution in 1:1 volume ratio. The ligand solution contained 0.1 M PbI_2_, 0.02 M PbBr_2_ and 0.04 M ammonium acetate in dimethylformamide (DMF). As soon as the solutions are in contact, they are mixed vigorously for 5 min (at room temperature). During this process the PbS QD are removed from octane to DMF phase and the ligand exchange is complete. The octane-OA phase is removed with the pipette and the QD are washed in fresh octane in order to remove the OA residues. After two washing steps, toluene is added to precipitate the QD, after which the QD are centrifuged and dried under vacuum for approx. 1 h and dispersed in butylamine so that the concentration is 200 mg/ml. This final solution is referred to as PbS QD ink. Next,1/4 of the ink was spin coated on top of the MgZnO layer at 1800 rpm for 30 s and immediately annealed at 70 °C for 10 min. This procedure gives 250 ± 10 nm thick samples. The 200 mg/ml solution was diluted with butylamine in 1:4 volume ratio to obtain a 50 mg/ml QD solution resulting in film thicknesses of 50 ± 10 nm. At this point the samples for PES are stored in darkness, in a desiccator before measurement. For the PES measurement the sample is mounted on a sample plate using a carbon tape and it is grounded by applying silver paint over the edge of the sample, connecting the surface of the sample with the sample plate.

### Material characterization

UV–Vis measurements were carried out with a double beam and monochromator Lambda 750 UV–Vis–NIR spectrophotometer with pre-aligned tungsten-halogen and deuterium source, and photomultiplier R955 and Peltier-cooled PbS as detectors. The wavelength range of the instrument is 190–3300 nm with wavelength accuracy of ± 0.15 nm for UV/Vis and ± 0.5 nm for NIR. Measurements of the thickness of the PbS thin films were made by means of a profilometer (Veeco Dektak 150).

### PES measurement

Soft X-ray photoelectron spectroscopy was carried out at the LowDosePES end-station at the PM4 beamline at BESSY II (Helmholtz-Zentrum Berlin, HZB)^37^, while the synchrotron was operated in single-bunch mode with a ring current of 15 mA. The beamline settings selected were 360 l/mm grating and cff = 1.6, the pressure in the analysis chamber was about 10^−9^ mbar and the sample was at ambient temperature. The analyser used for the detection of electrons was an angular-resolved time-of-flight spectrometer (ARTOF) with an acceptance cone of ± 15° for high photoelectron transmission. The raw data from the spectrometer was converted into intensity-versus-energy spectra using the IGOR ARTOF loader and analysis package available at the end-station.

The laser system installed at the beamline (Tangerine model from the company Amplitude Systèmes) was used at its second harmonic (515 nm), with a pulse length of about 350 fs, and a repetition frequency of 10.4 kHz for time-resolved PES measurements. For the spatial alignment of the X-rays and the laser spot on the sample, an yttrium–aluminum-garnet (YAG) crystal installed on the manipulator was used. An image of the laser on the crystal was recorded using a microscope camera and analysed in IGOR. The laser spot is an ellipse due to the 45° angle incidence and its size was analyzed through Gaussian fitting of the intensity profiles in the vertical and horizontal directions. The standard deviation of this Gaussian was 0.14 mm for the vertical axis and 0.25 mm for the horizontal axis. Thus, it was estimated that 68% of the laser power are incident in an area about 0.11 mm^2^. The power of the laser was adjusted by changing the efficiency of the external acousto-optic modulator of the laser system and was measured using a thermopile-based power sensor right before the in-vacuum laser incoupling optics. Two laser powers were used for measurements: 0.16 mW and 0.045 mW. These powers correspond to energies of 15.4 nJ and 4.3 nJ per pulse and the estimated laser fluences at the sample are: 9.5 μJ cm^−2^ and 2.7 μJ cm^−2^ per pulse. The X-ray spot size on the sample was estimated by a similar method to have a standard deviation of 0.065 mm for the vertical axis and of 0.14 mm for the horizontal axis.

Measurements with the laser were always carried out on fresh sample spots. Prior to each laser exposure, the sample was quickly analysed by an overview spectrum at multiple spots at the same photon energy as with the laser illumination confirming that different sample spots showed similar spectra. Measurements with the laser were carried out using a multiscan function developed by the beamline scientists to set up a sequence of several consecutive measurements in a fixed mode with the kinetic energy window centred on the core level of interest (Pb 5d in this study) and with the correct time-alignment of the laser and X-ray pulses. The temporal overlap between the laser and X-ray pulses was established by measuring the arrival times of the X-ray and laser photons using the ArTOF for photon detection. The laser delay was continuously electronically adjusted with respect to the X-rays.

Hard X-ray photoelectron spectroscopy (HAXPES) was carried out at the HAXPES end-station at the GALAXIES beamline of the SOLEIL French synchrotron facility (Paris)^50,51^ where the photon energy covers the 2.3–12 keV range. A photon energy of 3000 eV was selected using a Si (111) double-crystal monochromator. The photon flux was adjusted using the filters available at the beamline to avoid radiation damage of the samples. The samples were measured with a pressure of about 10^−8^ mbar in the analysis chamber. A high-resolution hemispherical electron analyser (SCIENTA EW4000) was used for detection of electrons with a pass energy of 200 eV and an analyser slit of 0.3 mm. Measurements of the Fermi edge and Au 4f. levels of a gold foil mounted on the manipulator were used for energy calibration for PES and HAXPES measurements.

## Supplementary Information

Supplementary Information.
