# Supplementary material for: A method for studying pico to microsecond time-resolved core-level spectroscopy used to investigate electron dynamics in quantum dots
Source: Sci Rep. 2020 Dec 31;10:22438. doi: 10.1038/s41598-020-79792-z (PMC7775430; doi:10.1038/s41598-020-79792-z)
Supplement: Supplementary file 1 — Supplementary Information. [file 41598_2020_79792_MOESM1_ESM.pdf]

Supplementary Information

A method for studying pico to microsecond time-resolved core-level spectroscopy used to investigate electron dynamics in quantum dots

Tamara Sloboda,[1] Sebastian Svanström,[2] Fredrik O. L. Johansson,[2] Aneta Andruszkiewicz,[3] Xiaoliang Zhang,[4] Erika Giangrisostomi,[5] Ruslan Ovsyannikov,[5] Alexander Föhlisch,[5,6] Svante Svensson,[2,7] Nils Mårtensson,[2,7] Erik M. J. Johansson,[3] Andreas Lindblad,[2] Håkan Rensmo,[2] Ute B. Cappel[1,*]

1 Division of Applied Physical Chemistry, Department of Chemistry, KTH Royal Institute of Technology, SE-100 44 Stockholm, Sweden

2 Division of Molecular and Condensed Matter Physics, Department of Physics and Astronomy, Uppsala University, Box 516, 751 20 Uppsala, Sweden

3 Department of Chemistry - Ångström Laboratory, Uppsala University, Box 523, 751 20 Uppsala, Sweden

4 School of Materials Science and Engineering, Beihang University, 100191 Beijing, China

5 Institute Methods and Instrumentation for Synchrotron Radiation Research, Helmholtz-Zentrum Berlin GmbH, Albert-Einstein-Straße 15, 12489 Berlin, Germany

6 Institute of Physics and Astronomy, University of Potsdam, Karl-Liebknecht-Straße 24/25, 14476 Potsdam, Germany

7 Uppsala-Berlin joint Laboratory on next generation photoelectron spectroscopy, Albert-Einstein-Str. 15, 12489 Berlin, Germany

* cappel@kth.se

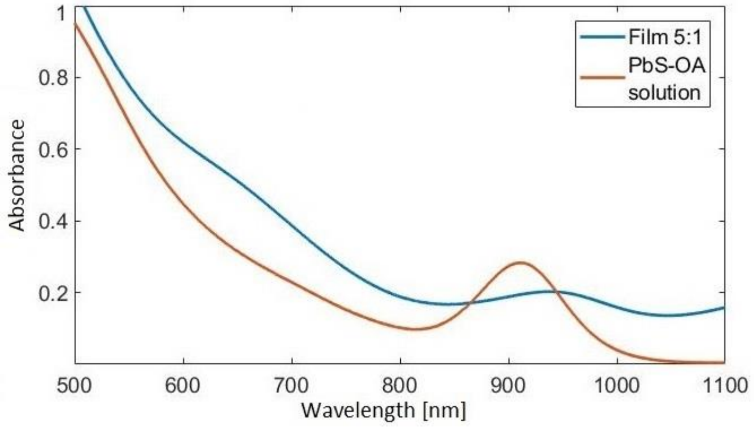

Figure S1. UV-visible absorption spectra of a PbS-OA solution (with the solvent spectrum subtracted) and of a 5:1 (250 nm) thin film PbS sample

Curve fitting of the steady-state photoelectron spectra

The spectra were fitted with a number of Voigt functions, representing the number of peaks present in the spectrum, and an appropriate background contribution (e.g. slope, polynomial, Shirley, Herrera

Gomez contribution). For the fit of the Pb 5d spectra, four peaks were used, representing two spin−orbit doublets (main and oxidized Pb). For I 4d and Br 3d, one spin−orbit doublet was used for fitting, whereas for the S 2p multiple spin orbit doublets were necessary to achieve the reasonable fit. The relative intensity and separation of all the doublets were fixed to those of the main doublet. Intensity ratio between Pb $4f_{7/2}$ and Pb $4f_{5/2}$, I $4d_{5/2}$ and I $4d_{3/2}$, and Br $3d_{5/2}$ and Br $3d_{3/2}$ peaks was fixed to 0.7±0.1, and the distance in BE to 4.8±0.1 eV. Intensity ratio between S $2p_{3/2}$ and S $2p_{1/2}$ was fixed to 0.5, and the distance in BE to 1.18 eV. The relative changes in peak positions and intensities could be determined from the fitted parameters.
For quantification of the HAXPES data, the intensities for each for level were divided by their ionization cross-section.[1] The final comparison is then done by dividing concentrations of the specific core levels to the most reliable one (in this case Pb 4f).

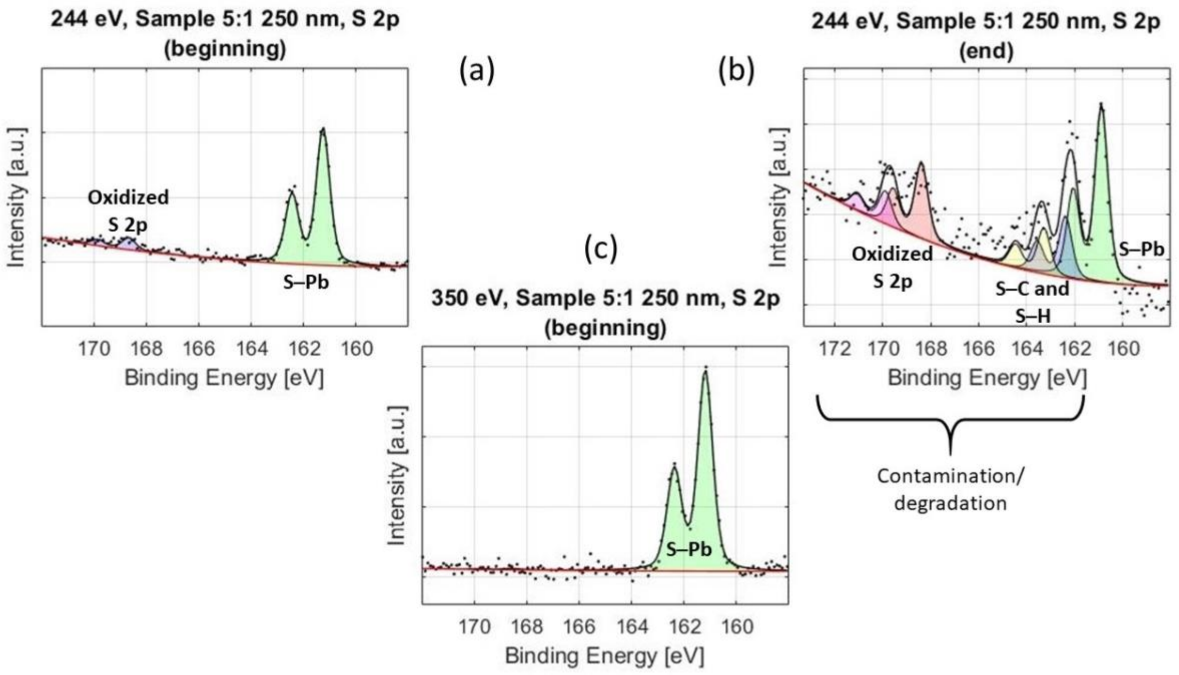

Figure S2. S 2p core level spectra measured at the LowDosePES end-station with curve fits included of a 250 nm thick PbS sample at 244 eV in the beginning (a) and in the end of an experiment (after being exposed to ambient atmosphere for a few days) (b) and at 350 eV in the beginning of an experiment (c). The fitting of the S 2p spectrum at 244 eV after being exposed to atmosphere for several days was more difficult due to noisy spectrum and there is a possibility of greater complexity of this spectrum

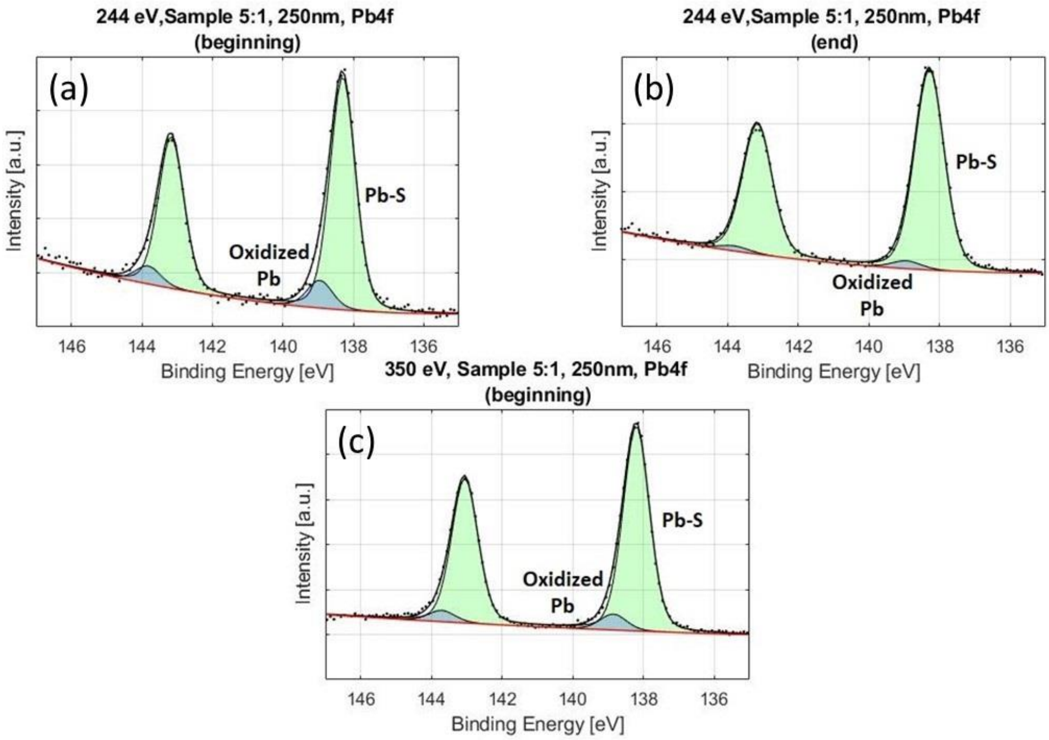

Figure S3. Pb 4f core level spectra measured at the LowDosePES endstation of the 250 nm thick PbS sample with curve fits included. The main Pb 4f doublet is assigned to Pb-S and Pb-I bonds, while the doublet at higher photon energy is assigned to Pb-O or Pb-Br bonds.; Sample 5:1 (250 nm) at 244 eV: fresh sample (a) and after exposure to ambient atmosphere for several days (b) and 350 eV of the fresh sample (c)

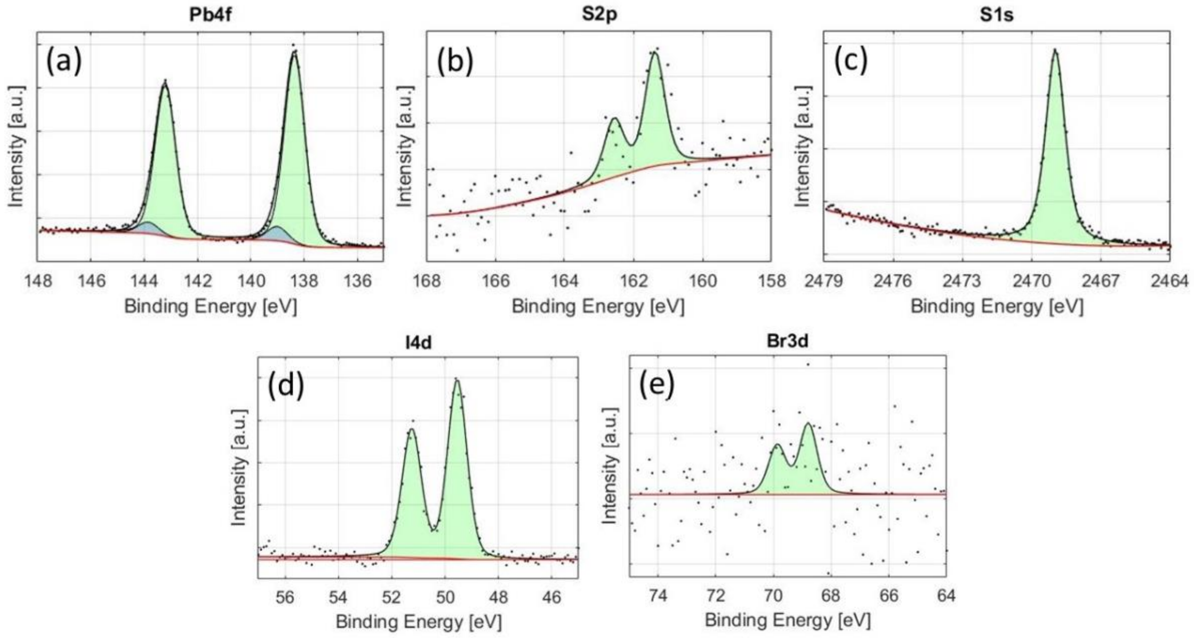

Figure S4. Spectra from HAXPES measurements at the Galaxies beamline of a 250 nm PbS sample at 3 keV with curve fits included. (a) Pb 4f, (b) S 2p, (c) S 1s, (d) I 4d and (e) Br 3d

Table S1. Fitting parameters of steady-state PES data measured at the LowDosePES endstation (data in Figures S2 and S3). R is the relative content of each element species in the fit.

5:1, 250 nm, 244 eV, fresh sample				
**R [%]**	**Peak**	**Position [eV]**	**Gaussian width [eV]**	**Lorentzian width [eV]**
**89.38**	Pb $4f_{7/2}$ (main)	138.30	0.77	0.14
**10.62**	Pb $4f_{7/2}$ (ox)	138.95	0.77	0.14
**92.03**	S $2p_{3/2}$ (main)	161.26	0.48	0.19
**7.97**	S $2p_{3/2}$ (ox)	168.66	0.48	0.19

5:1, 250 nm, 350 eV, fresh sample				
**92.77**	Pb $4f_{7/2}$ (main)	138.21	0.80	0.14
**7.23**	Pb $4f_{7/2}$ (ox)	138.86	0.80	0.14
**100**	S $2p_{3/2}$ (main)	161.19	0.56	0.19

5:1, 250 nm, 244 eV, after exposure to ambient atmosphere				
**96.23**	Pb $4f_{7/2}$ (main)	138.29	0.90	0.16
**3.77**	Pb $4f_{7/2}$ (ox)	138.94	0.90	0.16
**44.78**	S $2p_{3/2}$ (main)	160.88	0.53	0.19
**15.73**	S $2p_{3/2}$ (Pb-S-C)	162.38	0.53	0.19
**11.83**	S $2p_{3/2}$ (S-C, S-H)	163.28	0.53	0.19
**19.27**	S $2p_{3/2}$ (ox)	168.38	0.53	0.19
**8.40**	S $2p_{3/2}$ (ox)	169.88	0.53	0.19

Table S2. Quantification table and fitting parameters for HAXPES analysis of the sample 5:1 (data in Figure S4).

**Core level**	$^{X}/_{\mathbf{Pb4f(total)}}$	**Position [eV]**	**Gaussian width [eV]**	**Lorentzian width [eV]**
**Pb $4f_{7/2}$**	0.93	138.35	0.85	0.11
**Pb $4f_{7/2}$**	0.07	139.00	0.85	0.11
**I $4d_{5/2}$**	0.64	49.55	0.82	0.09
**Br $3d_{5/2}$**	0.13	68.79	0.67	0.11
**S $2p_{3/2}$**	0.63	161.36	0.67	0.14
**S 1s**	0.52	2468.98	0.86	0.31

Exponential fitting of kinetic traces

The final kinetic traces obtained at different laser powers and for samples with different thicknesses were fitted according to the following equation, where the first two exponentials describe the rise of the binding energy shift and the last three its decay:

$$\Delta E_B = -0.38\, a_2 e^{-\frac{t}{\tau_1}} - a_2 e^{-\frac{t}{\tau_2}} + a_3 e^{-\frac{t}{\tau_3}} + a_4 e^{-\frac{t}{\tau_4}} + a_5 e^{-\frac{t}{\tau_5}}$$

The amplitude of the first exponential was constrained to a_1=0.38 a_2, after this ratio was found for two data sets and was used to ensure the convergence of the fits for the other data sets.

The average rise ($\tau_{r,av}$) and decay times ($\tau_{d,av}$) were calculated from these parameters according to:

$$\tau_{r,av} = \frac{a_1\tau_1 + a_2\tau_2}{a_1 + a_2}$$

$$\tau_{d,av} = \frac{a_3\tau_3 + a_4\tau_4 + a_5\tau_5}{a_3 + a_4 + a_5}$$

Table S3: Parameters of multi-exponential fit of kinetic traces obtained for the 250 nm and the 50 nm thick PbS samples at laser pulse energies of 15.4 and 4.3 nJ.

PbS thickness / nm	250	250	50	50
Pulse energy / nJ	15.4	4.3	15.4	4.3
a_1 / eV	0.048	0.038	0.041	0.028
a_2 / eV	0.126	0.101	0.107	0.074
a_3 / eV	0.122	0.087	0.074	0.031
a_4 / eV	0.028	0.028	0.064	0.049
a_5 / eV	0.032	0.027	0.028	0.024
τ_1 / ns	0.43	1.5	0.61	1
τ_2 / ns	22	46	5.3	7.1
τ_3 / ns	470	890	110	140
τ_4 / ns	3,000	3,400	920	1,200
τ_5 / ns	28,000	28,000	24,000	24,000
$\tau_{r,av}$ / ns	15.7	33.7	4.0	5.4
$\tau_{d,av}$ / ns	5,800	6,500	4,400	6,300

References

1. Scofield, James H., L. L. L. *Theoretical Photoionization Cross Sections from 1 to 1500 keV*. **5**, (Livermore, Calif. : Lawrence Livermore Laboratory, University of California ; Springfield, Va., 1973).
